# Addressing the failures of undergraduate anatomy education: Dissecting the issue and innovating a solution

**DOI:** 10.1016/j.amsu.2020.12.024

**Published:** 2020-12-25

**Authors:** Karam Ahmad, Tahir Khaleeq, Umar Hanif, Nadia Ahmad

**Affiliations:** Royal Stoke University Hospital, Newcastle Road, Stoke-on-Trent, Staffordshire, ST4 6QG, UK

**Keywords:** Anatomy, Innovation, Surgical education, Training, Undergraduate teaching, History of medicine

## Abstract

**Background:**

Reduced time allocation, changes in teaching methods and Covid-19 have resulted in undergraduate anatomy teaching being marginalised.

This has implications on patient safety, litigation, student satisfaction and surgical workforce planning.

**Aims:**

The aim of this study is to survey a cohort of recent English medical graduates to attain their perspective on anatomy training and to propose an innovative solution to solve existing problems in undergraduate anatomy training.

**Methods:**

An online survey was sent out to 40 foundation doctors to offer insights into their undergraduate anatomy training. We asked participants to rate their perceived importance of anatomy, the importance offered to anatomy teaching at undergraduate level, preparation for clinical practice and future career plans.

**Results:**

22 participants responded to the online survey. All trained across England with equal spread between Northern and Southern medical schools. All participants perceived anatomy to be either important or very important in the survey. 20/22 felt that their undergraduate anatomy teaching was given very low to average importance by their institutions. 8/22 were confident or very confident with their anatomy knowledge on beginning clinical practice. Of the 22, 5 planned surgical careers, 10 did not know or gave other responses and 7 wanted to do General Practice. 16/22 said anatomy training had or will impact their decision on choosing a speciality.

**Conclusion:**

The current literature and above survey highlight the deficiencies that current doctors are facing.

We suggest implementation of a standardised anatomy curriculum and the development of an online anatomy course.

## Highlights

• The teaching of anatomy in medical schools is becoming increasingly marginalised.
• Reduced time, altered teaching methods and COVID-19 form the basis of this shift.
• This has implications on patient safety and surgical workforce planning.
• Literature and a nationwide survey highlight the inadequate nature of training.
• A standardised curriculum would benefit medical students and future surgeons.

## 1 Introduction

Long established as a key pillar of the medical curriculum, anatomy has survived the test of time. However, its relevance has significantly reduced across UK undergraduate teaching at medical school and indeed across the world [[1], [2], [3]]. The reduced focus on anatomy has directly impacted doctors in all settings but also results in surgically minded students not getting core exposure to this essential part of the curriculum.

Reasonings for this decline stem from several factors and over the past 30 years, the decline in teaching anatomy has been well documented across the undergraduate medical world [[4], [5], [6], [7], [8]]. Most commonly taught and examined in the ‘pre-clinical’ years of medical school, the required amount of anatomical knowledge required for most medical school finals is low, evidenced by the fact that 77% of medical schools use MCQs as a primary assessment tool [[9], [10], [11], [12]]. Most medical students will take up their foundation year one job with their last formal anatomy teaching session being 3–4 years prior, as up to 85% of medical schools teach anatomy in years one and two only [10]. The reduced focus on anatomy and lack of accountability, has led to the creation of a ‘tick box’ culture. This is evidenced by the fact that the average time spent studying anatomy in medical school is 149 hours over a five- or six-year course [3].

With regards to changing teaching methodologies, there appears to have been a shift in the UK towards a system based anatomical learning model as opposed to previous traditional regional methods [10]. Studies in 2003 suggested that students of the old system scored better than the ‘systems-based approach’ in standardised anatomy exams [11].

Other reasons for the decline of anatomy teaching include reduction in staffing, this is partially due to increasing pressures on juniors to not take time out of training which results in less availability for anatomy demonstrating. Furthermore, there has been a controversial shift away from learning by cadaveric dissection- long considered to be the gold standard of anatomical learning [12]. Running of the dissection rooms has become increasingly expensive and the needs to follow both national and European standards has meant significant challenges are encountered at each step of its functioning [13,14]. The current COVID 19 pandemic, has caused greater strain on this due to the need for social distancing.

Of note, attempts to define a ‘anatomy curriculum’ have been documented in studies but appear to have not reached the frontline of medical school training [15].

The results of these changes mean that there is genuine concern that the anatomical knowledge of junior doctors is ‘below acceptable levels’ and is undergoing an ‘alarming decline’ [11,[16], [17], [18], [19]]. The issue is prevalent across many developed nations, Australian medical students recently reported low confidence in their anatomical knowledge prior to graduation, a feeling which is not dissimilar to what is currently faced in the UK [20].

## 2 Aims

The aim of this study is to survey a cohort of recent English medical graduates to attain their perspective on anatomy training and to propose an innovative solution which will improve the current problems in undergraduate anatomy training.

## 3 Methodology

An online survey was sent to 40 randomly selected foundation year one and two doctors in the West Midlands region. The survey questions were provided by individual authors. For inclusion into the study, they required consensus of at least three authors. Disagreements were resolved by consensus. Participants who completed all the questionnaire were included, excluded were those who had graduated from medical school over 24 months ago, and those we did not go to a medical school in England. Participants were asked to state geographical location of their undergraduate studies and the year they completed medical school. Rating from *not very important* to *very important*, participants were asked how important they considered anatomy teaching and how much importance that they felt anatomy teaching was given at their institutions. We asked how confident (*Not very confident to very confident*) junior doctors were on starting their first post as doctors with regards to anatomy. We also asked participants to inform us of future speciality training plans and if their experience of anatomy teaching at undergraduate level had impacted their decisions for this.

## 4 Results

Of the 40 foundation doctors, 25 responded to the survey. Two were excluded due to graduating outside of England and one due to graduating over 24 months ago.

The average age of doctor was 24.6 with a range of 23–31. There was equal spread of medical schools from North and South England. Details of the survey questions and results can be found in Table .1.

**Table.1:** Survey questions and responses

Questions:	Responses	Results (/22)
Please can you rate your perceived importance of undergraduate anatomy teaching?	Very important	15
	Important	7
	Average	0
	Low importance	0
	Very low importance	0
How would you describe the importance given to undergraduate anatomy by your medical school?	Very important	0
	Important	0
	Average	6
	Low importance	6
	Very low importance	10
How would you describe your level of confidence in human anatomy when beginning your first rotation as a foundation year one doctor?	Very confident	1
	Confident	7
	Average confidence	4
	Not confident	6
	Not very confident	4
How would you rate your experience of anatomy?	Very Enjoyable	0
	Enjoyable	7
	Neutral	9
	Poor	5
	Very poor	1
Which subspeciality are you planning to pursue?	Any surgical speciality	5
	General Practice	7
	Other (not listed)	2
	Unsure/	8
Have your experiences of undergraduate anatomy influenced your decision on choosing a speciality?	Yes	16
	No	6

All participants rated anatomy teaching as either *important* or *very important*, with 15/22 rating it as the latter. However, almost all of the participants felt that undergraduate anatomy teaching was not given significant importance, with 20/22 feeling it was given *very low, low* or *average importance*. 10/22 felt it was given *very low importance.*

One participant was *very confident* with their anatomy knowledge on beginning clinical practice, seven were *confident*, and ten of the fourteen remaining were either *not very confident* or *not confident*. 7/22 stated that they *enjoyed* anatomy, and only 5 doctors planned surgical careers, 7 wanted to pursue a career in General Practice and the remaining were either unsure or chose other options. Of note, 16/22 said anatomy training has or will impact their decision on choosing a speciality.

## 5 Discussion

The above results and literature highlight several issues with anatomy teaching in England. First and foremost, these issues could have a direct impact on patient safety due to junior doctors’ inadequate knowledge. This increases risks of harm to patients, and subsequent litigation [21].

The lack of exposure to anatomy, places medical students with an interest in pursuing a surgical career at a clear disadvantage. This is often felt at the MRCS part A exam when many potential surgical trainees complain of having to learn new anatomy. The increasingly common trend of Core Surgical Trainees undergoing remedial anatomy revision in preparation for MRCS part B, either by courses or deanery teaching days is further evidence of this issue [6].

The lack of priority given to anatomy, may put medical students and therefore junior doctors of tomorrow in a false sense of security that i) anatomy is not essential to becoming a competent doctor, but also ii) that only surgeons need to have a complex understanding of anatomy. Quite to the contrary, with the advances made in other specialities, in particular interventional radiology and cardiology, anatomy has become even more relevant to the non-surgical doctor. The above survey results only serve to highlight this further, as most doctors did not feel confident with their anatomical knowledge on starting their first jobs.

Finally, historically surgery has been known to be amongst the most competitive specialities in medicine. Since the turn of the decade, it is now commonplace for unfilled surgical posts to be advertised long after application windows closing [22]. Perhaps some of this can be attributed to the lack of exposure at an undergraduate level to surgically focussed anatomy. Increasing the focus on anatomy, could help improve what could be an upcoming shortage of surgeons and support workforce planning.

The issues above may suggest that anatomy has not modernized in its learning techniques. Even today, the didactic style of teaching forms the basis of learning. Attempts to modernise anatomy are still in their infancy, and recent studies have shown there to be benefit in the use of interactive lecture-based dissection halls [23] and virtual reality in cardiac anatomy teaching [24].

We propose the development of a nationally standardised anatomy curriculum. This as mentioned earlier has already been established [15] but needs wider adoption by medical schools. This would ensure a minimum standard of anatomical knowledge amongst English graduates. For the surgically inclined, we would propose a collaboration with the Royal College of Surgeons in order to develop and innovate a modular anatomy course, which enables students, including the surgically inclined medical student, to optionally enrol and complete a nationally standardised program. Initially independent of medical schools, this would allow us to make use of 21st century technology and modernise anatomy. In particular, modernisation could occur via the use of anatomy apps which could include written presentations, videos and augmented reality-based dissection. This can be taught to the level of MRCS Part A, which puts more medical students on the road to a potential surgical career. In the current Covid-19 pandemic, where social distancing can reduce numbers of students and demonstrators in the dissection room, a program like this could be extremely beneficial.

The standardised approach will ensure that all doctors, regardless of their potential subspecialist interest will be able to call upon a uniform basic understanding of anatomy.

The optional anatomy course would enable surgically inclined students to start building their surgical portfolio, prepare them for the MRCS examinations and increase their exposure to anatomy. Beyond students who want to pursue a surgical career, it also enables those who are unsure about future options to be given an insight in to anatomy, more so than the average of 149 hours over 5 years that they currently get [10].

Importantly, the use of apps, augmented reality and e-modules mixed with the traditional prosection/dissection approach offered by current medical schools ensures that anatomical teaching is current, relevant (especially in current times) and modernized.

### 5.1 Limitations

The limitations of this study stem from the small sample size, the lack of representation from all medical schools in England, the lack of objective measurement in the survey and the use of foundation doctors who would have had their anatomy teaching experience several years ago.

The study was affected by the novel Covid-19 virus hence was altered to meet Government guidelines. This resulted in a decision to involve only foundation doctors as the pandemic led to closures of medical schools and changes in anatomy curriculum delivery. The advantage of this was that the study focusses on the impact undergraduate anatomy training has on clinical work as a foundation doctor-the primary end goal of any undergraduate anatomy teaching program.

There are two potential drawbacks of the proposal.

Firstly, development of the anatomical curriculum would be dependent on the General Medical Council (GMC) as they are the standard setting organisation for medical schools and doctors. Implementation would have to undergo a thorough process to ensure quality, whilst this process is essential, this can take a significant period of time and be costly.

Collaboration with the Royal Colleges of Surgeons would require extensive time from senior surgeons/anatomists to set and evaluate the contents for the course. Permission would be needed from current material owners to allow integration into modules. In addition, development of new material would be needed. Again, drawbacks of this are related to both time, cost of development and support infrastructures once set up.

### 5.2 Future work

Looking ahead, it would be prudent to address some of the limitations of this study. We would recommend a larger sample size, ideally in a post Covid environment where we can assess the impact this has had on training. We would also recommend using medical students as part of the study, an undergraduate exit survey could be proposed at the end of the foundation program job application for example. To objectively assess anatomy performance once could consider an exam similar to the ‘Prescribing Safety Assessment (PSA)’ for all final year medical students to take. This, however, does not take clinical correlation as a doctor into consideration.

## 6 Conclusion

In conclusion, it is clear that there is a widespread belief that undergraduate teaching of anatomy is inadequate, outdated and does not benefit those students who may be considering, or who have chosen to pursue a surgical career. This is unfair to both our students and our patients. It is an issue which needs addressing sooner rather than later, for the sake of workforce planning. Whilst one cannot blame the entire shortage of surgeons down to poor anatomy education at undergraduate level, it is perhaps most sensible to start making changes at the most junior stages of our future doctor's careers to make surgery attractive. The changes discussed above, we believe, are sensible, achievable and would ensure that anatomy continues to be a mainstay of the medical curriculum.

## 7 Provenance and peer review

Not commissioned, externally peer reviewed.

## Ethical approval

No ethical approval was required.

## Funding

No funding was required.

## Author contribution

Karam Ahmad: Conception, design, review of literature, drafting and final approval of the paper.

Tahir Khaleeq: Data collection, analysis, drafting and final approval of the paper.

Umar Hanif: Data collection, analysis, drafting and final approval of the paper.

Nadia Ahmad: Data collection, analysis and final approval of the paper.

## Annals of medicine and surgery

The following information is required for submission. Please note that failure to respond to these questions/statements will mean your submission will be returned. If you have nothing to declare in any of these categories then this should be stated.

## Please state any sources of funding for your research

None.

## Consent

N/A.

## Registration of research studies

1 Name of the registry: Research Registry.
2 Unique Identifying number or registration ID: researchregistry6287.
3 Hyperlink to your specific registration (must be publicly accessible and will be checked): https://www.researchregistry.com/register-now#home/registrationdetails/5fbce8ae7b3bbc001bdd6f77/

## Guarantor

Karam Ahmad, Tahir Khaleeq, Umar Hanif, Nadia Ahmad.

## Declaration of competing interest

None.
